# Interferon-Stimulated Gene 15 Conjugation Stimulates Hepatitis B Virus Production Independent of Type I Interferon Signaling Pathway* In Vitro*

**DOI:** 10.1155/2016/7417648

**Published:** 2016-10-27

**Authors:** Yujia Li, Shilin Li, Xiaoqiong Duan, Yanzhao Chen, Baihai Jiao, Haiyan Ye, Min Yao, Limin Chen

**Affiliations:** ^1^Institute of Blood Transfusion, Chinese Academy of Medical Sciences and Peking Union Medical College, Key Laboratory for Transfusion-Transmitted Diseases of Sichuan Province, Chengdu, Sichuan 610052, China; ^2^Toronto General Research Institute, University of Toronto, Toronto, ON, Canada M5G 1L6

## Abstract

Hepatitis B virus (HBV) is an important account of infectious hepatitis and interferon (IFN) remains one of the best treatment options. Activation of type I IFN signaling pathway leads to expressions of IFN-stimulated genes (ISGs) which play important roles in antiviral and immunomodulatory responses to HBV or hepatitis C virus (HCV) infection. Our previous studies indicated that ISG15 and its conjugation (ISGylation) were exploited by HCV to benefit its replication and persistent infection. This study was designed to assess the role of ISG15 and ISGylation in HBV infection* in vitro*. The levels of ISG15 and ISGylation were upregulated by ISG15 plasmid transfection into HepG2.2.15 cells. Decreased ISGylation was achieved by siRNA targeting UBE1L, the only E1 activating enzyme for ISGylation. Overexpression of ISG15 and subsequent ISGylation significantly increased the levels of HBV DNA in the culture supernatants although the intracellular viral replication remained unaffected. Silencing UBE1L, with decreased ISGylation achieved, abrogated this ISGylation-mediated promoting effect. Our data indicated that overexpression of ISG15 stimulated HBV production in an ISGylation-dependent manner. Identification of ISG15-conjugated proteins (either HBV viral or host proteins) may reveal promising candidates for further antiviral drug development.

## 1. Introduction

Hepatitis B virus (HBV) is a major cause of liver disease with approximately 400 million people infected all over the world [1]. Up to one million deaths caused by liver cirrhosis and primary hepatocellular carcinoma (HCC) following chronic HBV infection makes it a serious public health problem. Unfortunately, current type I interferon- (IFN-) based antiviral therapy has suboptimal antiviral effect [2, 3], indicating that substantial drug resistance mechanism should be explored in order to develop more effective treatment strategies.

More than 300 IFN-stimulated genes (ISGs) can be induced by type I IFNs [4], and these ISGs play different roles: direct or indirect antiviral activity, cytoskeletal remodeling, apoptosis induction, posttranscriptional and posttranslational regulation, viral molecules detection, and signaling pathways modulation [5–7]. ISG15, a structural homology to ubiquitin [8], is one of the most abundant IFN-induced ISGs. Similar to ubiquitin, ISG15 can conjugate to target proteins, a process called ISGylation, which is catalyzed by E1 activating (UBE1L), E2 conjugating (UbcH6 and UbcH8), and E3 ligase (Herc5). Ubiquitin-like specific protease 18 (USP18) is the specific deconjugating enzyme that removes ISG15 from its targets [9].

The antiviral activity of ISG15 (and ISGylation) has been verified in a lot of studies investigating the virus and host interactions in either the ISG15/Herc5 overexpression/knockdown cells or ISG15/USP18 deficient mice (reviewed in [5, 7, 10]). Quite interestingly, studies from our group and others demonstrated that increased hepatic baseline (pretreatment) expression levels of a subset of ISGs, including ISG15 and USP18, were associated with treatment nonresponse to type I IFN-based therapy in both HCV [11–13] and HBV [14] patients. ISGs have been classically known as antiviral, but why increased ISGs contribute to treatment failure remains obscure. However, it has been revealed that that ISG15 (and ISGylation) not only stimulated HCV production but also blunted IFN anti-HCV activity either in HCV replicon cells or in cells infected with hepatitis C virus [15, 16]. These results indicate that HCV exploits ISG15 as a host immune evasion mechanism to facilitate its replication and persistence.

In this study, we set out to investigate the effect of ISG15 (and ISGylation) on HBV replication and production. ISG15 (and ISGylation) expression levels were modified by ISG15 overexpression plasmid or siRNA targeting UBE1L. Our results indicated that ISG15 promotes HBV production dependent on ISGylation. This study provides novel evidence that HBV exploits host innate immunity to facilitate its persistence.

## 2. Methods

### 2.1. ISG15 Expression Plasmid and Cell Culture

As previously described [15], human full-length ISG15 gene was generated using pOTB7-ISG15 plasmid DNA (MGC clones; Open Biosystems) as template, and the resulting PCR product was cloned into a pcDNA4/HisMax TOPO TA expression vector (Invitrogen, USA). The sequences of the primers are 5′-ATGGGCTGGGACCTGACGGTG-3′ (forward) and 5′-TTAGCTCCGCCCGCCAGGCTC-3′ (reverse), respectively. Plasmid Maxiprep Kit (Qiagen, German) was used to prepare plasmid DNA for transfection.

HepG2.2.15 cells with integrated full-length HBV genome were kindly provided by Professor Bo Qin (Chongqing Medical University, China), as previously described [17], and were cultured in Dulbecco's Modified Eagle's Medium (DMEM) (Gibco, USA), supplemented with 10% heat-inactivated fetal bovine serum (Gibco, USA), 100 IU mL^−1^ penicillin (Gibco, USA), 100 IU mL^−1^ streptomycin (Gibco, USA), and 1 mg mL^−1^ G418 (Invitrogen, USA) in 5% CO_2_ at 37°C.

### 2.2. ISG15 Transfection and Confirmation of ISG15 Expression

Twenty-four hours before transfection, HepG2.2.15 cells were seeded in 6-well plates at 6 × 10^5^ cells per well with antibiotics-free medium. Transfection complexes were prepared in 400 *μ*L serum-free medium by mixing 4 *μ*g (unless otherwise indicated) plasmid DNA and 12 *μ*L polyethyleneimine (PEI, 1 *μ*g *μ*L^−1^) and then letting the mixture stand for 15 minutes at room temperature. Cells were washed three times with antibiotics-free medium and transfection mix was added into each well containing 2 mL antibiotics-free medium and the culture medium was changed 12 hours later. Two days following transfection, both cells and medium were harvested.

Total RNA was prepared using TRIzol (Invitrogen, USA) and reverse-transcription was carried out using iScript cDNA Synthesis Kit (BIO-RAD, USA) according to the manufacturer's protocol. The resulting cDNA was amplified by Faststart Universal SYBR Green Master Mix (Roche, USA). Cycle parameters were 94°C, 10 min; (94°C, 45 s; 56°C, 45 s; 72°C, 1 min) ×30 cycles; 72°C, 5 min. ISG15 and glyceraldehyde-3-phosphate dehydrogenase (GAPDH) primers for quantitative PCR were listed in Table 1. Western blot, the process described in detail previously [15], was also performed for further confirmation of ISG15 protein expression and ISGylation.

### 2.3. UBE1L Silencing to Inhibit ISGylation

siRNA targeting human UBE1L (siGENOME SMARTpool, M-019759-00-0005), negative control siRNA (siGENOME Non-Targeting siRNA Pool #2, D-001910-02-05), and Transfection Reagent (DharmaFECT 4, T-2004-02) were purchased from GE Dharmacon, USA. HepG2.2.15 cells were plated in 24-well plates at a density of 1 × 10^5^ per well overnight. Media were changed and the cells were transfected in solution with siRNA at a final concentration of 50 nM according to the protocol. Twenty-four hours later, 1.4 *μ*g per well ISG15 plasmid was transfected into the cells. Total RNA, cellular proteins, and supernatant DNA were prepared 48 h after transfection to evaluate the silencing efficiency and HBV production.

### 2.4. Quantification of HBV Production

For HBV production quantification, culture supernatant DNA and total intracellular RNA were extracted using QIAamp DNA Blood Mini Kit (Qiagen, German) and TRIzol (Invitrogen, USA) according to kit manufacturer's instructions, respectively. Real-time PCR was carried out as stated above with the HBV-specific primers (Table 1). Intracellular covalently closed circular DNA (cccDNA) was extracted and purified according to a previous report [18] measured by a real-time PCR described previously [19]. Supernatant HBsAg and HBeAg were detected by enzyme-linked immunosorbent assay (ELISA) kits (Biosamite, Shanghai, China) following the manufacturer's protocols. Intracellular HBcAg expression was assessed by western blot by using a polyclonal anti-HBcAg antibody (Boster Biological Technology, Wuhan, China) and integrated densities were calculated using ImageJ software.

### 2.5. Statistical Analyses

Difference between two categorical values was compared by Student's* t*-test. Statistical significance was set at *P* < 0.05. The experiments were repeated at least three times.

## 3. Results

### 3.1. Increasing ISG15 Expression and ISGylation in HepG2.2.15

**Figure 1:**
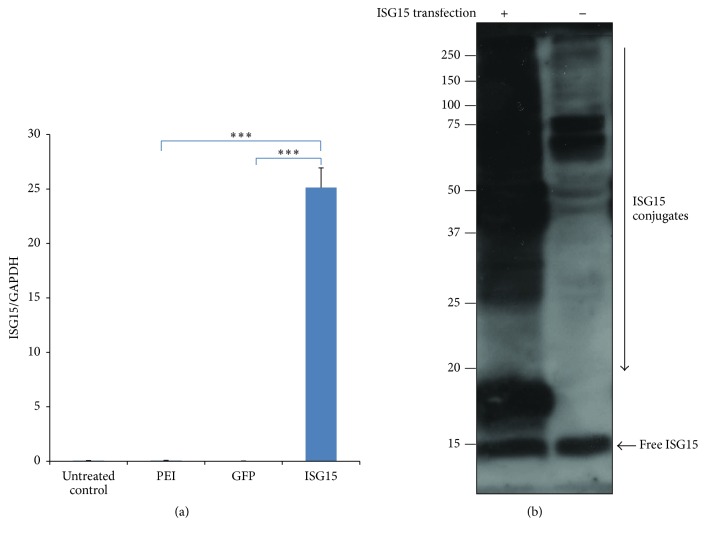
*ISG15 expression and ISGylation were increased by transfection*. HepG2.2.15 cells were transfected with the ISG15 expression plasmid or GFP-expressing plasmid. (a) Levels of ISG15 mRNA were determined by real-time PCR (normalized by GAPDH) 24 hours after transfection. PEI, transfection regent polyethyleneimine (PEI) treatment only; GFP, transfected with 4 *μ*g GFP-expressing plasmid; ISG15, transfected with 4 *μ*g ISG15 plasmid. The results are presented as the means ± SD, *n* ≥ 3; error bars indicate SD. ^*∗∗∗*^ *P* < 0.001. (b) Protein ISGylation was further assessed by western blot with or without ISG15 overexpression. Molecular mass markers are shown on the left (kDa).

As the antiviral effect of ISG15 is mainly attributed to its forming ISGylated proteins, we asked whether ISG15 conjugation can be increased by ectopic expression of ISG15. As seen in Figure 1(a), transfection of ISG15 led to a pronounced increase in ISG15 expression which was further confirmed by western blot (Figure 1(b)). In addition, unlike HeLa cells, in which ISGylation is difficult to be induced [20], overexpression of ISG15 alone increased ISGylation (Figure 1(b)) in HepG2.2.15 cells.

### 3.2. ISG15 Promotes HBV Production* In Vitro*

**Figure 2:**
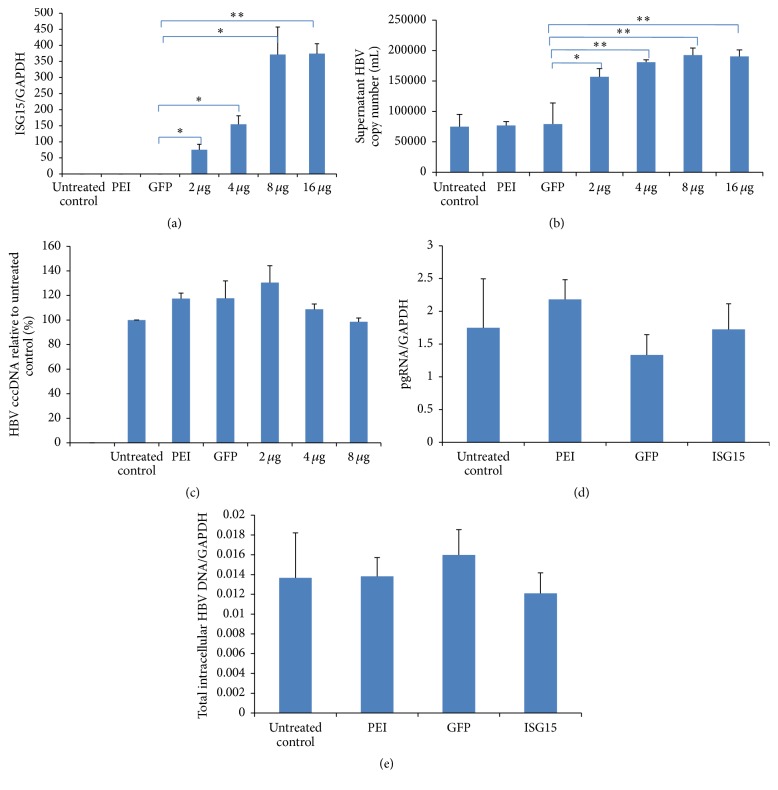
*ISG15 overexpression promotes HBV production in vitro*. HepG2.2.15 cells were transfected with the indicated amount of ISG15 plasmid or GFP-expressing plasmid. (a) Real-time PCR showing ISG15 mRNA expression 24 h after transfection of ISG15 plasmid. GFP, transfected with 4 *μ*g GFP-expressing plasmid; 2 *μ*g, 4 *μ*g, 8 *μ*g, or 16 *μ*g transfected with 2 *μ*g, 4 *μ*g, 8 *μ*g, or 16 *μ*g ISG15 plasmid. (b) Supernatant HBV DNA, intracellular, (c) cccDNA, (d) pgRNA, and (e) total HBV DNA were determined by real-time PCR 48 h after transfection, respectively. PEI, transfection regent polyethyleneimine (PEI) treatment only; GFP, transfected with 4 *μ*g GFP-expressing plasmid; ISG15, transfected with 4 *μ*g ISG15 plasmid. The results are presented as the means ± SD, *n* ≥ 3; error bars indicate SD. ^*∗*^ *P* < 0.05; ^*∗∗*^ *P* < 0.01.

**Figure 3:**
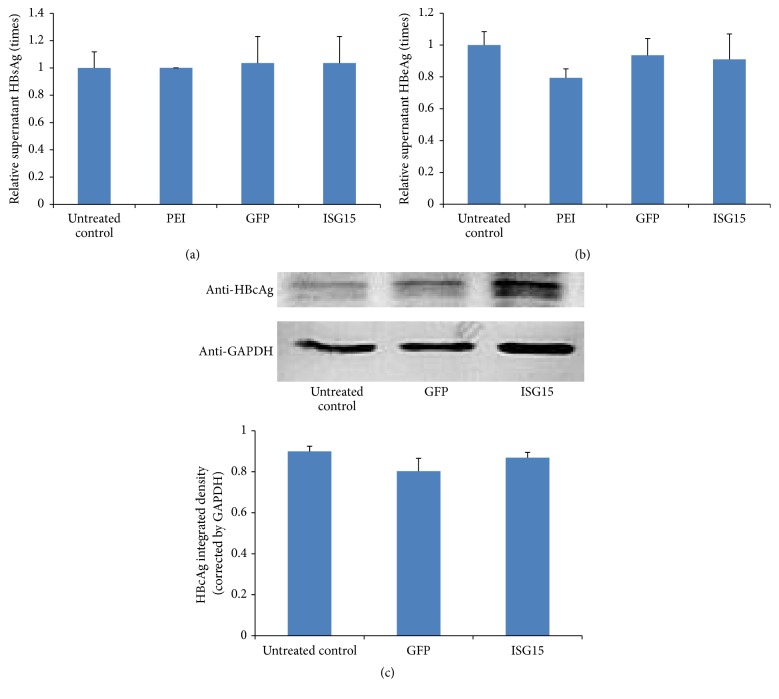
*HBV protein expression after ISG15 overexpression*. HepG2.2.15 cells were transfected with the ISG15 plasmid or GFP-expressing plasmid. (a) HBsAg and (b) HBeAg in the culture medium were detected by ELISA, respectively. (c) Intracellular HBcAg was assessed by western blot. Relative integrated density was calculated by ImageJ software and normalized by GAPDH expression. PEI, transfection regent polyethyleneimine (PEI) treatment only; GFP, transfected with 4 *μ*g GFP-expressing plasmid; ISG15, transfected with 4 *μ*g ISG15 plasmid. The results are presented as the means ± SD, *n* ≥ 3; error bars indicate SD.

To evaluate the effects of ISG15/ISGylation on HBV replication and production, we measured the intracellular total HBV DNA, covalently closed circular DNA (cccDNA) HBV pgRNA levels, and total HBV DNA in the supernatant of cell cultures, following ISG15 overexpression in HepG2.2.15 by real-time PCR. The results showed that although the intracellular viral cccDNA (Figure 2(c)), pgRNA (Figure 2(d)), and total HBV DNA (Figure 2(e)) seemed not affected by ISG15 overexpression and ISGylation, the supernatant HBV DNA was obviously increased in a ISG15 dose-dependent manner (Figures 2(a) and 2(b)). Since dead cells, cell debris, and apoptotic bodies may also be responsible for the elevated HBV DNA in the medium, we monitored HBV replication by further measuring the supernatant HBsAg (Figure 3(a)) and HBeAg (Figure 3(b)) using ELISA kits and cellular HBcAg (Figure 3(c)) using western blot. However, none of these proteins expressions was significantly affected by elevated ISG15 and ISGylation.

### 3.3. Silencing UBE1L Inhibits ISGylation and Abrogates the Promoting Effect of Overexpressed ISG15 on HBV Production

**Figure 4:**
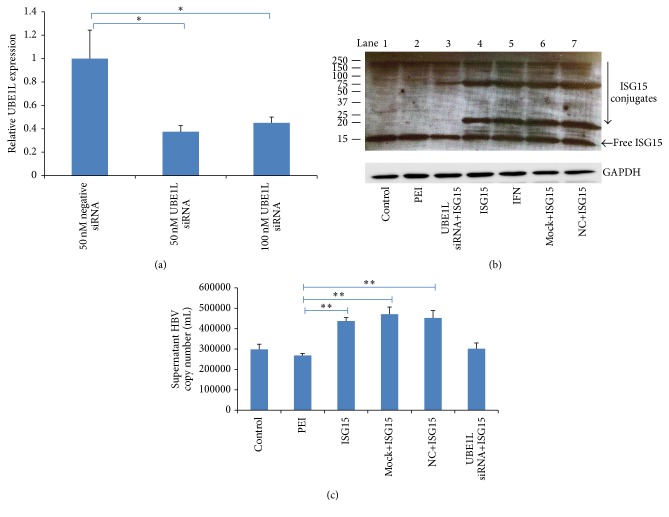
*ISGylation is important in ISG15-promoted HBV production*. UBE1L knockdown was performed by RNAi to abrogate ISGylation in HepG2.2.15 cells. (a) Knockdown efficiency was determined by real-time PCR showing UBE1L mRNA expression 24 h after 50 nM negative siRNA, 50 nM UBE1L siRNA, or 100 nM UBE1L siRNA treatment. (b) Comparison of protein ISGylation (western blot for ISG15) after UBE1L knockdown and ISG15 overexpression. Molecular mass markers are shown on the left (kDa). (c) Real-time PCR was used to assess the supernatant HBV DNA after UBE1L knockdown and ISG15 overexpression. Control, untreated control; PEI, ISG15 transfection regent polyethyleneimine (PEI) treatment only; ISG15, ISG15 overexpression by transfection with 1.4 *μ*g ISG15 plasmid; UBE1L siRNA+ISG15, 50 nM UBE1L siRNA treatment followed by ISG15 overexpression; Mock+ISG15, siRNA transfection regent treatment followed by ISG15 overexpression; NC+ISG15, 50 nM negative siRNA treatment followed by ISG15 overexpression; IFN, 100 IU/mL IFN*α*2b treatment. The results are presented as the means ± SD, *n* ≥ 3; error bars indicate SD. ^*∗*^ *P* < 0.05; ^*∗∗*^ *P* < 0.01.

Though mature form of ISG15 conjugation to target proteins was shown to be essential for ISG15-dependent antiviral effects, both free ISG15 (intracellular or extracellular) and ISGylation could be detected [9], indicating potential functions of free ISG15 involved in viral infection. We thereby investigated whether free form ISG15 or the ISGylation was responsible for the increased HBV production. Since UBE1L catalyzes the adenylation of ISG15, inhibition of UBE1L can suppress ISG15 conjugation. First of all, we determined the efficiency of UBE1L knockdown by real-time PCR. As shown in Figure 4(a), UBE1L mRNA expression was significantly inhibited by 50 nM or 100 nM UBE1L siRNA. We then used 50 nM UBE1L siRNA in the following experiment. UBE1L knockdown was further confirmed by western blot for ISG15 (Figure 4(b)). Compared with the nonknockdown groups (Figure 4(b), lanes 4, 6, and 7), UBE1L-knockdown cells showed lower level of ISG15-modified proteins (Figure 4(b), lane 3) in response to ISG15 overexpression, although the levels of free ISG15 experienced no apparent difference. IFN*α*2b treated HepG2.2.15 cells were included as positive control to show the expression of ISG15 and ISGylation (Figure 4(b), lane 5). In parallel with UBE1L knockdown and consequent downregulation of ISGylation, the supernatant HBV DNA returned to baseline level (Figure 4(c)). These results indicated that ISG15 stimulated HBV production in an ISGylation-dependent manner.

We then asked whether UBE1L knockdown itself could inhibit HBV production or not. We suppressed UBE1L expression in HepG2.2.15 cells without overexpression of ISG15 and monitored the HBV production by measuring intracellular total HBV DNA, pgRNA, and HBcAg, as well as HBV DNA, HBsAg, and HBeAg in the culture medium. Interestingly, HBV production seemed not affected by UBE1L knockdown (see Supplementary Figures 1-2 in Supplementary Material available online at http://dx.doi.org/10.1155/2016/7417648).

### 3.4. ISG15 Promotes HBV Production Independent of Type I IFN Signaling Pathway

**Figure 5:**
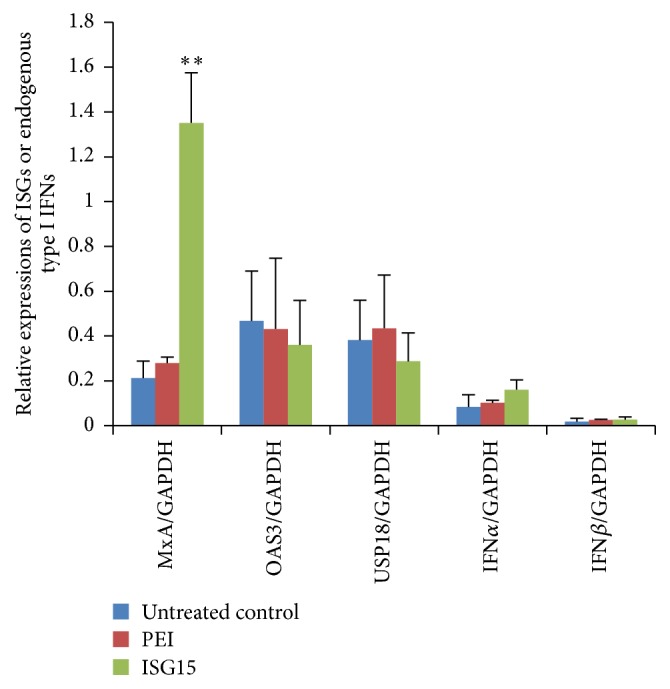
*Effects of ISG15 overexpression on ISGs and endogenous type I IFNs*. HepG2.2.15 cells were transfected with ISG15 plasmid. Real-time PCR was performed to quantify MxA, OAS3, USP18, IFN*α*, and IFN*β* 48 h after transfection. PEI, transfection regent polyethyleneimine (PEI) treatment only; ISG15, transfected with 4 *μ*g ISG15. ^*∗∗*^ *P* < 0.01.

As PEG-IFN*α* is one of the efficient agents approved for the treatment of chronic HBV, we then investigated whether overexpression of ISG15 boosted HBV production through blocking endogenous type I IFN production or inhibiting activation of type I IFN signaling pathway. Our results showed that although elevated ISG15/ISGylation was parallel with increased expression of ISGs such as MxA (myxovirus resistance 1) in HepG2.2.15 cells, other ISGs like OAS3 (2′-5′-oligoadenylate synthetase 3) and USP18 and endogenous expressions of type I IFN were not affected significantly (Figure 5). In addition, HBV production in HepG2.2.15 cells was scarcely affected by short-period (48–72 h) IFN*α*2b treatment (our unpublished data). These results suggested that ISG15 may be involved in HBV production independent of type I IFN signaling pathway.

## 4. Discussion

Although ISG15 exerts its function in three different forms including free intracellular ISG15, conjugated ISG15, and extracellular ISG15 as a cytokine, ISGylation is the undoubtedly important one. ISG15 is able to conjugate with over 300 cellular proteins [21–23] and many viral proteins {e.g., influenza A virus NS1 protein [24] and human papillomavirus (HPV) L1 capsid protein [25]}, directly altering the functions of the modified proteins or disrupting the ubiquitination process to prevent degradation of the conjugated proteins. In our study, blocking ISGylation abrogated the increased production of HBV, indicating that ISGylation is the predominant form of ISG15 to boost HBV production in HepG2.2.15 cells. Due to this fact, we also checked the antiviral effects of UBE1L by suppressing UBE1L alone (without ISG15 overexpression) in HepG2.2.15 cells. Unfortunately, no significant anti-HBV effect was observed. Since ISG15 is an IFN-stimulated gene which is mainly induced by IFN stimulation, the baseline expression of ISG15 in HepG2.2.15 is limited. This may help us to explain, at least partly, why knockdown UBE1L could not significantly inhibit HBV production in HepG2.2.15.

Although ISG15/ISGylation has been proposed as an efficient host defense response to various viral infections, increasing evidence implied this antiviral mechanism may be virus-specific and ISG15/ISGylation may be exploited by some viruses to facilitate viral replication and production by either negatively regulating host immunity [26, 27] or directly promoting viral replication through blunting IFN antiviral activity [15, 28].

In many respects, ISGylation/deISGylation process resembles ubiquitination/deubiquitination system which is involved in most physiological and pathological processes, suggesting the potential regulation activity of ISG15/ISGylation in biology, especially in innate immunity. ISGylation of the proteins involved in IFN signaling may inhibit the signaling cascade. RNA helicase enzymes retinoic acid inducible gene I (RIG-I) is a pattern recognition receptor (PRR) which can be activated by viral infection, resulting in endogenous type I IFN expression. Kim et al. [26] demonstrated that ISGylation of RIG-I can reduce both basal and virus-induced IFN promoter activity at the cellular level in a negative feedback manner. Jeon et al. [29] found that ISGylation of filamin B antagonizes the type I IFN-induced Jun N-terminal kinase (JNK) signaling pathway. Filamin B acts as a scaffold that tethers the cascade members in type I IFNs activated JNK-specific signaling cascade which is composed of RAC1, MEKK1, MKK4, and JNK. ISG15 conjugation of filamin B leads to the release of RAC1, MEKK1, and MKK4 from the scaffold protein, interdicting the signaling. Consequently, it is easy to understand why decreased ISGylation results in an increased phenotypic sensitivity to IFN-*α* in several hepatoma derived cell lines or even nonhepatic cell lines [16, 30]. Our previous work [15] also indicated that ISGylation promotes HCV production partly by decreasing the anti-HCV effect of IFN-*α*. We therefore explored the expressions of type I interferon and IFN pathway activation in HepG2.2.15 cells. Consistent with previous studies [31, 32] that have demonstrated that ISG15 could increase phosphorylation of JAK2, STAT1, and IRF-3, promoting type I IFN signaling pathway, our results also revealed an activated type I IFN signaling characterized by amplified expressions of some ISGs. However, no significant change of IFN*α* or IFN*β* expression was observed following ISG15 overexpression. Although it has been reported [33] that HBV replication can be significantly suppressed in HepG2.2.15 cells with the 9-day treatment of novel liver-targeting interferon *α*2b (IFN-CSP), our data (unpublished) showed that HBV production in HepG2.2.15 cells is not sensitive to short-period IFN*α*2b treatment (e.g., 24–72 h treatment), which indicated that the effects of transient ISG15 upregulation (and ISGylation) on HBV production might be independent of type I IFN. Stable transfected HepG2.2.15 cells may be employed to investigate the effect of ISG15 (and ISGylation) on type I IFN signaling pathway in future study.

On the other hand, considering the apparent antiviral role of ISG15, it is not surprising that many viruses have developed immune evasion tactics during evolution to avoid ISGylation by either inhibiting the formation of conjugation or disrupting the formed modification. As reported by Yuan and Krug [34], NS1 of influenza B virus can noncovalently bind to ISG15 and inhibit interaction between ISG15 and UBE1L, thereby inhibiting ISGylation. Guerra et al. [35] also observed that vaccinia virus E3 protein can bind to ISG15 through its C-terminal domain and inhibit ISG15 conjugation. Some other viruses, like Dugbe virus (DUGV) and SARS coronavirus, can even hydrolyze ISG15 from target proteins by their ovarian tumor domain- (OTU-) containing proteases [36] or papain-like proteases [37]. Moreover, in the present study, it seems that HBV has evolved to make use of ISGylation to promote its production, indicating a novel mechanism for HBV persistence. In order to explore the detailed mechanism, we used real-time PCR (as described in [38–41]) to test the expression levels of sodium taurocholate cotransporting polypeptide (NTCP), VSP4B, and 78 kDa glucose-regulated protein (GRP78), all of which have been identified as essential factors in HBV life cycle [42, 43]. However, none of these genes experienced conspicuous changes in mRNA expressions following overexpression of ISG15 (data not shown). Therefore, we hypothesize that, instead of regulation of gene expression, ISGylation may primarily alter the function or the stability of cellular or viral proteins involved in HBV production, especially in HBV secretion considering the unaffected intracellular pgRNA, cccDNA, and total HBV DNA. Our study reveals the complexity and multiple roles of ISGylation, and further work will be needed to determine the precise mechanism, so that therapies targeting these pathways or targets can be developed in the future.

More interestingly, the function of ISG15 (and ISGylation) during viral infection seems to be restricted to specific hosts. Kim et al. [44] reported that HBV replication was not affected in ISGylation-deficient mice. They constructed a HBV replication model by injecting a replication-competent DNA construct hydrodynamically into the mice. Although there is a significant difference in ISG15 conjugation between UBE1L knockout mice and wild mice, HBV replication was not affected. Studies of influenza B virus may provide some implication to explain the difference; although NS1 of influenza B virus can protect virus from ISGylation by binding ISG15 in human primates [34], it cannot bind to mouse ISG15 [45], indicating the species-specific feature of ISG15/ISGylation.

In conclusion, in this current study we reported an important and novel role of ISG15 and ISGylation in regulating HBV life cycle in HepG2.2.15 cell model stably expressing HBV. Results from this study provide a rational explanation for drug (especially type I IFN) resistance and persistent infection of HBV, making ISG15 a possible predictor to traditional IFN therapy and a potential antiviral target for HBV treatment.

## Supplementary Material

The following is the description of Supplementary Material:Taken into account that our data showed that ectopic ISGylation promoted HBV production, the suppression of UBE1L was then checked for its potential anti-HBV effects. We knockdown UBE1L by RNAi to abrogate baseline ISGylation in HepG2.2.15 cells which was further confirmed by real-time PCR for UBE1L mRNA expression 24h after siRNA treatment (Supplement Figure 1A). HBV production was evaluated by a series of in vitro assays. Quantitative PCR was applied to determine HBV DNA in the culture media, intracellular pgRNA and total HBV DNA (Supplement Figure 1B-D). ELISA and western blot were employed to investigate the supernatant HBsAg, HBeAg (Supplement Figure 2A-B) and intracellular HBcAg (Supplement Figure 2C), respectively. However, our results indicated that HBV production was not significantly affected by suppression of UBE1L alone in HepG2.2.15 cell line. Since ISG15 is a IFN-stimulated gene which is mainly induced by IFN stimulation, the baseline expression of ISG15 and ISGylation in HepG2.2.15 is limited. This may help us to explain, at least partly, why UBE1L suppression itself could not significantly inhibit HBV production in HepG2.2.15 cells.

## Figures and Tables

**Table 1:** Primers used for real-time PCR.

	Nucleotide sequence
ISG15	F: 5′-CGCAGATCACCCAGAAGATT-3′
	R: 5′-GCCCTTGTTATTCCTCACCA-3′
GAPDH	F: 5′-GCCTCCTGCACCACCAACTG-3′
	R: 5′-ACGCCTGCTTCACCACCTTC-3′
HBV	F: 5′-CGTTTTTGCCTTCTGACTTCTTTC-3′
	R: 5′-ATAGGATAGGGGCATTTGGTGGTC-3′
HBV pgRNA	F: 5′-CTCAATCTCGGGAATCTCAATGT-3′
	R: 5′-TGGATAAAACCTAGCAGGCATAAT-3′
MxA	F: 5′-GTGCATTGCAGAAGGTCAGA-3′
	R: 5′-CTGGTGATAGGCCATCAGGT-3′
OAS3	F: 5′-GAATTCTCCCATCAAAGTGATCAA-3′
	R: 5′-CTCAGATGCCGACCTCGTGGT-3′
USP18	F: 5′- CAGACCCTGACAATCCACCT-3′
	R: 5′- AGCTCATACTGCCCTCCAGA-3′
IFN *α*	F: 5′-TCGCCCTTTGCTTTACTGAT-3′
	R: 5′-GGGTCTCAGGGAGATCACAG-3′
IFN *β*	F: 5′-AAACTC ATAGCAGTCTGCA-3′
	R: 5′-AGGAGATCTTCAGTTTCGGAGG-3′

pgRNA, pregenomic RNA; MxA, myxovirus resistance 1; OAS3, 2′-5′-oligoadenylate synthetase 3; USP18, Ubiquitin specific protease 18.
