# Supplementary material for: Interferon-Stimulated Gene 15 Conjugation Stimulates Hepatitis B Virus Production Independent of Type I Interferon Signaling Pathway In Vitro
Source: Mediators Inflamm. 2016 Oct 27;2016:7417648. doi: 10.1155/2016/7417648 (PMC5102744; doi:10.1155/2016/7417648)

Supplement Figure 1. Effects of UBE1L suppression on HBV production in HepG2.2.15 cells. UBE1L knockdown was performed by RNAi to abrogate baseline ISGylation in HepG2.2.15 cells. **(A)** Knockdown efficiency was determined by real-time PCR showing UBE1L mRNA expression 24h after 50nM negative siRNA or 50nM UBE1L siRNA treatment. **(B)** Supernatant HBV DNA, intracellular **(C)** pgRNA and **(D)** total HBV DNA were determined by real-time PCR at 48h or 72h after UBE1L siRNA transfection. Mock, siRNA transfection regent treatment only; 50nM negative siRNA, transfected with 50 nM negative siRNA; 50nM UBE1L siRNA, transfected with 50 nM UBE1L siRNA. The results are presented as the means ±SD, n⩾ 3, error bars indicate SD.

Supplement Figure 2. Effects of UBE1L suppression on HBV protein expression in HepG2.2.15 cells. UBE1L knockdown was performed by RNAi to abrogate baseline ISGylation in HepG2.2.15 cells. Culture medium was collected 48h after to detect **(A)** HBsAg and **(B)** HBeAg by ELISA, respectively. Total protein was collected at 48h or 72h after UBE1L siRNA transfection to assess **(C)** intracellular HBcAg by western blot. Mock, siRNA transfection regent treatment only; negative siRNA, transfected with 50 nM negative siRNA; UBE1L siRNA, transfected with 50 nM UBE1L siRNA. The results are presented as the means ±SD, n⩾ 3, error bars indicate SD.

Supplement Figure 1

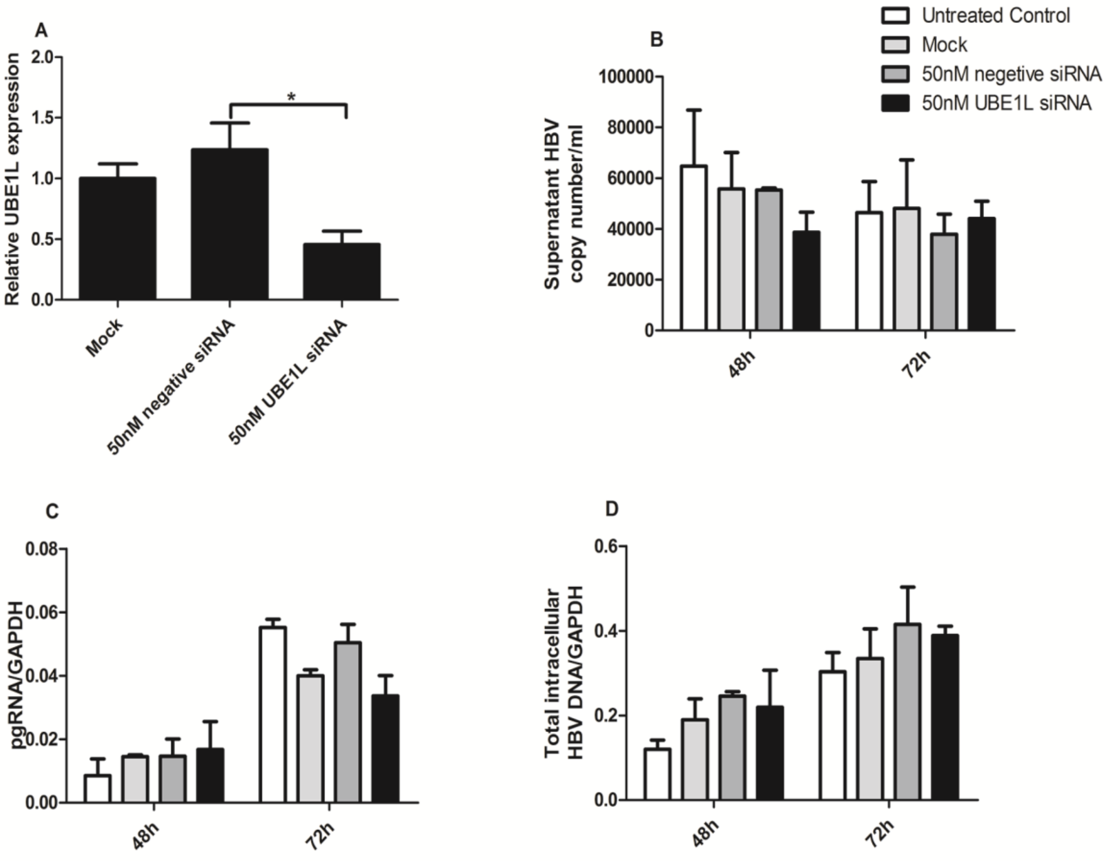

Supplement Figure 2

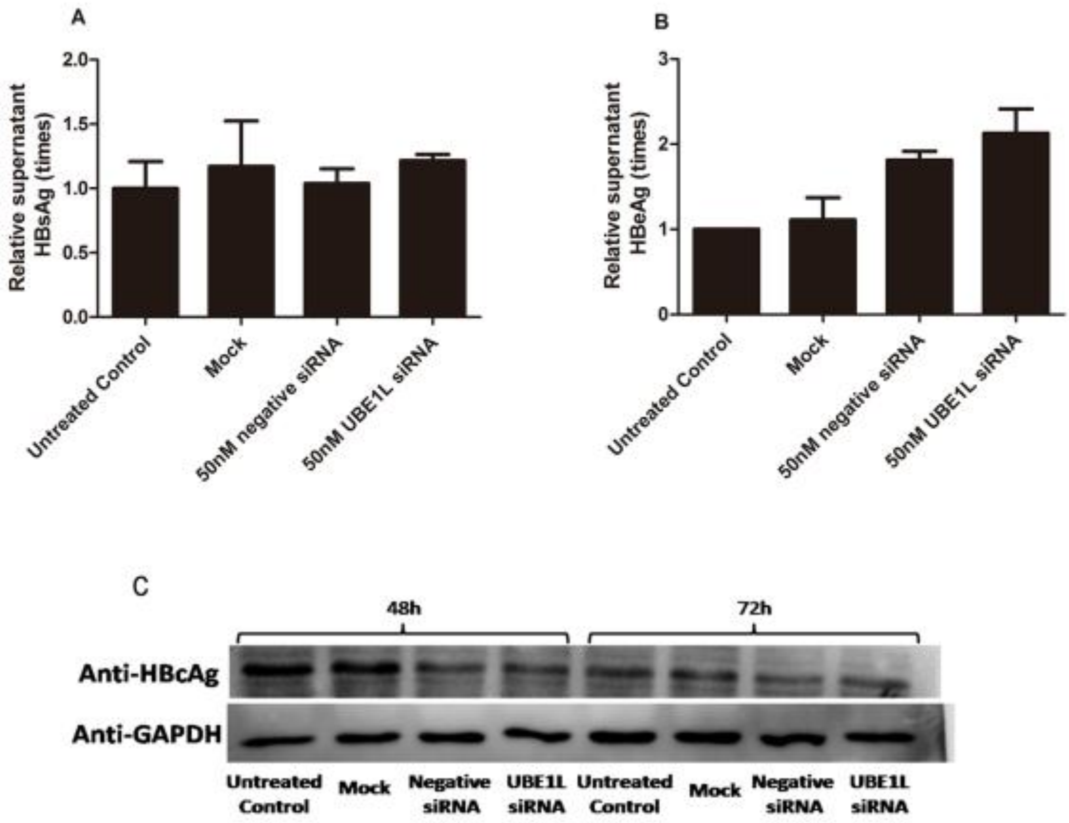

Supplement: Supplementary file 1 — The following is the description of Supplementary Material:Taken into account that our data showed that ectopic ISGylation promoted HBV production, the suppression of UBE1L was then checked for its potential anti-HBV effects. We knockdown UBE1L by RNAi to abrogate baseline ISGylation in HepG2.2.15 cells which was further confirmed by real-time PCR for UBE1L mRNA expression 24h after siRNA treatment (Supplement Figure 1A). HBV production was evaluated by a series of in vitro assays. Quantitative PCR was applied to determine HBV DNA in the culture media, intracellular pgRNA and total HBV DNA (Supplement Figure 1B-D). ELISA and western blot were employed to investigate the supernatant HBsAg, HBeAg (Supplement Figure 2A-B) and intracellular HBcAg (Supplement Figure 2C), respectively. However, our results indicated that HBV production was not significantly affected by suppression of UBE1L alone in HepG2.2.15 cell line. Since ISG15 is a IFN-stimulated gene which is mainly induced by IFN stimulation, the baseline expression of ISG15 and ISGylation in HepG2.2.15 is limited. This may help us to explain, at least partly, why UBE1L suppression itself could not significantly inhibit HBV production in HepG2.2.15 cells. [file 7417648.f1.pdf]
